# Evaluation of the Accero Stent for Stent-Assisted Coiling of Unruptured Wide-Necked Intracranial Aneurysm Treatment with Short-Term Follow-Up

**DOI:** 10.3390/jcm9092808

**Published:** 2020-08-31

**Authors:** Wojciech Poncyljusz, Kinga Kubiak, Leszek Sagan, Bartosz Limanówka, Katarzyna Kołaczyk

**Affiliations:** 1 Department of Diagnostic Imaging and Interventional Radiology, Pomeranian Medical University, 71-252 Szczecin, Poland; wojciech.poncyljusz@pum.edu.pl (W.P.); kolaczyk@radiologia.szczecin.pl (K.K.); 2 Department of Neurosurgery and Pediatric Neurosurgery, Pomeranian Medical University, 71-252 Szczecin, Poland; leszekm.sagan@gmail.com (L.S.); blimanowka@gmail.com (B.L.)

**Keywords:** Accero, cerebral aneurysm, neuroradiology, embolization, endovascular treatment

## Abstract

Background: Stent-assisted coiling is an effective method of treating intracranial aneurysms. The aim of the study was to assess the safety and efficacy of the new Accero stent for the treatment of intracranial aneurysms. Materials and Methods: It was a retrospective, single-center study. Eighteen unruptured intracranial aneurysms were treated using the stent-assisted coiling method with the Accero stent. Patient demographics, aneurysm characteristics, procedural parameters, grade of occlusion, complications, and clinical results were analyzed. Follow-up magnetic resonance (MR) was performed 6 months after intervention. Results: Seventeen patients with 18 incidental unruptured aneurysms were electively treated with coiling and the Accero stent. The aneurysms were located on internal carotid artery (ICA), middle cerebral artery (MCA) and basilar artery (BA). All stents were deployed successfully. Immediate complete occlusion rate Raymond-Roy occlusion classification (RROC) class I was achieved in 13 cases and class II in 4 cases. Complications occurred in 2/17 treatments and included guidewire stent perforation with subarachnoid hemorrhage (SAH) and stent deformation. Vascular spasm in the subarachnoid hemorrhage (SAH) patient subsided before discharge. Ninety days after intervention, the modified Rankin Scale (mRS) value was 0. RROC class I was observed in 88.23% of cases in follow-up. Conclusion: The Accero stent provides excellent support for coil mass. It constitutes an efficacious device with good initial occlusion rate for treating wide-necked unruptured intracranial aneurysms.

## 1. Introduction

Endovascular coiling therapy and stent-assisted coiling (SAC) is an alternative to open neurosurgical treatment and constitutes a well-established modality for the treatment of intracranial aneurysms [1,2,3,4]. In addition, a large group of flowdiverters and new non-stent systems, which help treat broad-necked aneurysms (such as Woven EndoBridge (WEB) or PulseRider), are available commercially [5,6,7]. However, stents remain the largest and most commonly used group by neuroradiologists. Stents are used, mostly, for the security of coil protrusion into the parent artery, coil stability in the aneurysm sac, and increasing the packing density [2,8,9]. They also possess other qualities, e.g., they cause a partial change in arterial geometry that causes flow vector to change within the aneurysm [10]. Stents are also safer than flowdiverters in terms of thrombo-embolic complications [7]. There are different constructions and materials used among available stents for SAC, hence the differences in appearance and behavior in the vascular environment [3,11,12,13]. Stents were designed for broad-necked aneurysms, but wide-necked aneurysms at arterial bifurcation and large curves, e.g., in the internal carotid artery syphon, constitute the most difficult challenges for them. The goal of this preliminary report was to assess the feasibility, safety, and effectiveness of the new Accero stent (Acandis GmbH, Pforzheim, Germany) and to analyze short term follow-up when it is used for treating wide-necked intracranial aneurysms.

## 2. Materials and Methods

It was an observational and a retrospective single-center study that included a series of wide-necked unruptured intracranial aneurysms. We included all patients with unruptured wide necked (neck ≥ 4 mm or dome to neck ratio < 2) brain aneurysms (<20 mm maximum diameter in any plane) between 18 and 75 years. We excluded patients with significant extra or intracranial stenosis of the parent artery, with contraindications to the use of antiplatelet agents, with high risk of embolic stroke, and those patients who were unable to complete the required follow-up. All cases were treated in 2019 with the Accero stent-assisted coiling by the same operator. The study protocol was approved by the Bioethics Committee no. KB-0012/29/05/2020/Z. Informed consent for the procedure was obtained from all patients. We collected clinical and radiological data prospectively, which were then retrospectively reviewed. Demographics, aneurysm-characteristics, procedural parameters, the grade of occlusion, complications, and the clinical results were analyzed. All procedures were performed under general anesthesia. Acetylsalicylic acid in a dose of 150 mg/daily and clopidogrel in a dose of 75 mg/daily was administered for 7 days before the treatment and continued for 3 months after discharge. The 6F Brite Tip sheath introducer (Cordis, Bridgewater, NJ, USA) was placed with transfemoral percutaneous access, and the guiding catheter Chaperon 6F (MicroVention-Terumo, Tustin, CA, USA) was positioned in the C1 segment of the internal carotid artery (ICA). Digital subtraction angiography (DSA) and 3D-DSA were performed before treatment and after DSA and flat panel computed tomography (Vaso-CT). Reconstruction in all cases was done on Philips (Azurion Clarity IQ-Medical Systems Nederland BV). The Traxcess 0.014-inch microguidewire (MicroVention-Terumo, Tustin, CA, USA) and Headway 17 microcatheter (MicroVention-Terumo, Tustin, CA, USA) were used under 3D Roadmap for the deployment of all Accero stents. In all cases, the jailing technique was used finally. After stent implantation, coil embolization was performed. Stent selection was performed in accordance with the manufacturer’s instructions and the largest size of the aneurysm dome. After stent implantation, coil embolization was performed using 18 or 10 platinum 360 or 3D and then helical coils (MicroVention-Terumo, Tustin, CA, USA). The degree of aneurysm occlusion was evaluated using the Raymond-Roy occlusion classification (RROC) [14]. Clinical evaluation was performed using the modified Rankin Scale (mRS) by a neurosurgeon before the procedure, after the embolization, and at discharge.

The femoral artery puncture site was always closed with the FemoSeal (Terumo Europe) vascular closure system.

## 3. Results

**Figure 1:**
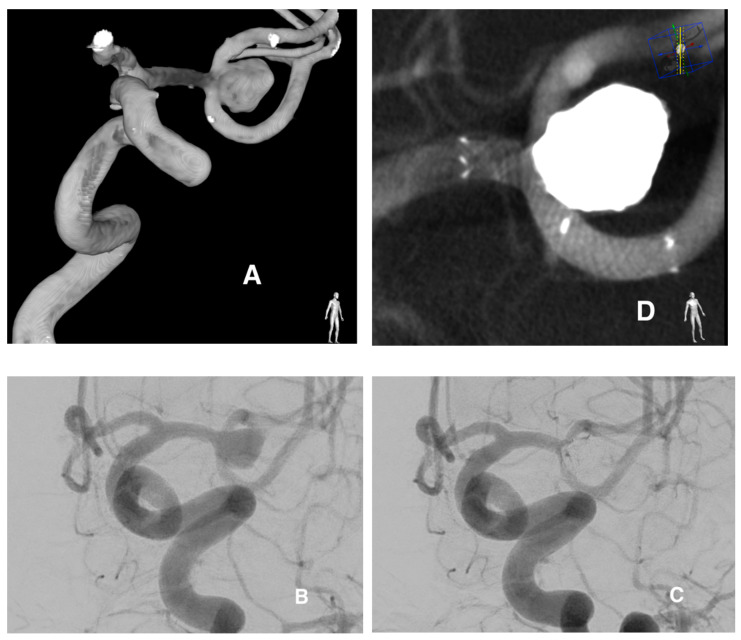
Wide-necked left middle cerebral artery (MCA) bifurcation aneurysm visible on 3D reconstruction (**A**) and digital subtraction angiography (DSA) in working projection (**B**) demonstrates the complex morphology of the lesion. The aneurysm was treated with the stent-assisted coiling using the Accero device which is shown on post-treatment DSA (**C**). Intraprocedural contrast-enhanced flat panel detector computed tomography (CT) (MIP reconstruction) showing complete occlusion of the aneurysm and (**D**) full stent opening with excellent opposition to the tortuous segment of the vessel which can be seen thanks to highly visible enhanced radiopacity of the platinum-nitinol composite wire and stent markers.

Seventeen patients with 18 incidental unruptured aneurysms were treated with stent-assisted coiling with the Accero stent device. Patient and aneurysm characteristics are presented in Table 1. A total of 64.7% of the patients were female and 35.3% male, with a mean age of 63 years (range 42–70 years). There was no atherosclerosis in the treated vessels. The aneurysms were located in the internal carotid artery (58.8%), the middle cerebral artery (35.3%) and the basilar artery (5.9%). All aneurysms were wide-necked (dome-to-neck ratio of ≤ 2), 7 (41.2%) cases were bifurcation aneurysms, and 10 (58.8%) were sidewall aneurysms. The mean dome size was 8 mm, and the mean value for the aneurysm neck was 4.5 mm. Pre-treatment mRS was 0. Accero stents were deployed successfully in all cases with good wall apposition, which did not require percutaneous transluminal angioplasty (PTA) to be performed inside the stent, except in one case. All stents in various configurations were characterized by excellent visibility, due to radiopacity of the Platinum-Nitinol composite wire, which allowed the visualization of the entire contour of the stent (Figure 1). Three additional platinum markers at each end plus the middle marker facilitated stent positioning under the aneurysm neck significantly. The immediate complete occlusion rate RROC class I was achieved in 13 cases (76.47%) and RROC class II in 4 cases (23.52%) (Table 2). Complications occurred in 2/17 procedures (11.7%), including the perforation due to the stent’s pushing wire (directly related to the procedure) with SAH (*n* = 1) and stent deformation (*n* = 1). No case of stent thrombosis was observed. Perforation occurred due to trauma incited by the long stent’s pushing wire of small aneurysm located above the treated aneurysm. Consequently, the patient developed a small SAH and vascular spasm with neurological symptoms. However, the symptoms subsided almost completely until the discharge from the hospital (mRS 1). Stent deformation occurred during the treatment of an aneurysm of the ICA that exhibited complicated anatomy of the syphon. Incomplete opening at the proximal segment of the stent was noticed after implantation. PTA with Eclipse 2L balloon (Balt, Montmorency, France) was performed, which slightly changed the structure of the stent, and eventually it was not hemodynamically relevant. There were no clinical complications or puncture site adverse events. Ninety days after the procedure, mRS values were 0. Six-month follow-up magnetic resonance angiography angiography revealed that complete occlusion occurred in 15 cases (88.23%) (class RROC I). Hypertension and nicotinism had no statistically significant effect on treatment outcomes (*p* value > 0.05).

## 4. Discussion

Nowadays, neuro-endovascular techniques are the first-line therapy for the majority of intracranial aneurysms [15,16], becoming an alternative method for surgical clipping. Endovascular approach of non-ruptured aneurysms (ATENA) showed that endovascular treatment of unruptured intracranial aneurysms was associated with low morbidity and mortality rates. Transient or permanent neurological deficits occurs in 5.4% of the 649 patients (1000 aneurysms), with one-month morbidity and mortality of 1.7% and 1.4% [17]. Brinjikji et al. showed that patients (65–79 years old) treated with coiling had significantly lower morbidity (6.9% vs. 26.8%) and mortality (0.8% vs. 2.0%) rates compared to patients who underwent clipping. In addition, patients aged 80 years or older who underwent coiling had lower morbidity (9.8% vs. 33.5%) and mortality (2.4% vs. 21.4%) compared to clipping [18]. The safety and efficacy of stent-assisted coiling have been demonstrated in numerous studies [1,2,3,4]. It was proved that SAC provides better immediate and long-term occlusion rate compared with coiling only [1,2]. This technique prevents coil protrusion or dislocation into the parent artery by increasing the density of coil packing, and accelerates endothelialization [1,2,19,20]. Moreover, an important feature is that SAC increases treatment procedure safety in complex and broad-necked aneurysms. Equipment manufacturers are struggling to produce a stent that will be safer, easier to use, and that is going to provide an excellent long-term clinical result for the treatment of aneurysms. Currently, a large number of different stents are available commercially, such as laser-cut stents in open-cell or closed-cell design, as well as braided stents [3,11,12,13]. All these systems have different advantages and disadvantages. Braided stents have become more practical, as they can be used in a wider range of complicated cases and they exhibit less disadvantageous behavior when compared to open-cell stents that cause a significant increase in the cell size at the outer curvature of tortuous vessels [11,12,13,14]. Laser-cut stents with closed-cell design can kink in tight arterial bends or ovalize crossing curves on different vessel diameter [13]. Furthermore, the lack of re-sheathing, which is necessary in cases with high vessel tortuosity, constitutes a significant disadvantage of such stents, which results in increased tension being exerted on the equipment, and can cause dislocation during deployment [8,19]. From the operator’s point of view, the essential features include the stent’s excellent visibility during the procedure, its flexibility and easy adaptation to the curvatures of vascular anatomy, and good adherence to the vessel wall, all of which reduce the risk of clotting and accelerate endothelialization. The Accero stent is a new, highly visible, self-expanding braided stent designed for SAC. It differs from other stents in that it has advantages such as different radial force, very smooth surface manufactured using the BlueXide technology in order to optimize hemocompatibility, very small cell size reminiscent of flowdivert devices, and enhanced radiopacity of the platinum-nitinol composite wire. Other braided stents available on the market such as LVIS, LVIS Jr. or all LEO+ and Baby LEO+ almost all contain nitinol wire and they exhibit different behavior and visualization [3,11,12,13,16]. There is one newer stent, LVIS EVO (MicroVention-Terumo, Tustin, CA, USA), that was launched on the market in 2020, but no publication regarding it has been published yet. Our own experience shows that both stents, Accero and LVIS Evo, behave in a similar way, however Accero is slightly stiffer and has a much longer pushing guidewire. It can be embarrassing because it is far from the field of observation during the implantation of the stent and can cause injury to the small vessel if you do not pay attention to it, which happened to us with Accero stent in one case (5.9%) in which perforation with SAH occurred. Aside from this technical complication in this study, all Accero stents were deployed successfully in all treatment procedures and also exhibited good apposition to the vessel wall in cases with high tortuosity of vascular anatomy, which is similar to the reported findings of another study [21].

No collapse or flattening was observed. The control of the pushing guide wire was lost beyond the significantly enlarged image of the implantation site in only two cases. This experience resulted in the use of smaller magnifications in the following cases with Accero. Another interesting feature may be the fact that the density of the Accero stent constitutes an advantage in the majority of cases giving the features of a flowdivert, but in some, it can be disadvantageous, as was observed by us: attempts at passing a microcatheter through the dense stent mesh were not successful in 5 cases, which is a disadvantage. Therefore, we recommend the jailing technique with the Accero stents. In one case (5.9%) the stent did not open completely proximally, but this feature is also observed for other well-known brain stents and can be corrected in most clinical cases [3,11,12,13,22,23,24]. The incidence of technical and clinical complications is similar to other braided stents [3,22,23,24,25]. No procedure-related stroke occurred in our series, but this observation may be ascribed to the patient group being relatively small. However, these complications may depend on the type of stent and must be checked on a larger group. Also, other various complications were reported, depending on the type of stent used [3,11,12,13,22,23,24,25,26]. The metal coverage of the Accero stent depends on the size of the implanted stent and parent vessel diameter to 19%. It is significantly larger than laser-cut stents and other braided stents, except the LVIS EVO stent, in which metal coverage is relatively high: up to 28% [26,27]. Accero and LVIS EVO stents can offer certain flow-diverting effects, which is an advantage, but we used Accero with coils in all cases, and we cannot ascertain this precisely.

In this study, the immediate complete occlusion rate RROC class I complete obliteration was 13 (76.47%), which is consistent with the immediate SAC occlusion results described in the literature [4,24]. The meta-analyses of Phan et al. and Hong et al. reported that the value for immediate occlusion rate for SAC was 57.7% (range: 20.2–89.2%) [4,24]. The better metal coverage of the Accero stent may also result in a change in the intra-aneurysmal flow occlusion, which was also reported [19]. Occlusion improvement can be expected in follow-up [3,21,25].

We can only report 6-month MR follow-up; however, the treatment was highly efficient, as complete occlusion occurred in 88% (15 cases), which is similar to other reports regarding the cases treated with Accero [21].

## 5. Limitations

This study has some limitations. Firstly, the number of patients in this study was small. This is a single-center study with only six months of MR follow-up, but the data were collected prospectively. All procedures were performed by highly experienced interventional neuroradiologists who have implanted a large number of all types of stents for over 15 years, so it can be assumed that the final result would not be significantly different in terms of the frequency of technical and clinical complications. Only the stent was introduced and the number of reports were limited, so the main focus of the investigation was to evaluate the safety and effectiveness of the Accero stent.

## 6. Conclusions

The Accero stent provides excellent support of coil mass and constitutes an effective and safe device with good initial occlusion rate when used for the treatment of wide-neck unruptured intracranial aneurysms. Further studies are needed to assess its long-term effectiveness.

## Figures and Tables

**Table 1:** Patient and aneurysm characteristics.

Characteristic			
**Age**	61.4 ± 9.0		
	(42–70)		
**Sex**	Female	Male	
	11 (64.7%)	6 (35.3%)	
**Aneurysm location**	ICA	MCA	BA
	10 (58.5%)	6 (35.3%)	1 (5.9%)
**Aneurysm size**	5.1 ± 4.2 mm		
**Aneurysm configuration**	Sidewall saccular		Bifurcation saccular
	10 (58.8%)		7 (41.2%)
**Neck diameter**	3.9 ± 2.1 mm		
**Proximal and distal parent vessel diameter**	Maximum: 4 mm		Minimum: 1.5 mm
**Maximum difference between distal and proximal parent vessel diameter**			
**Smoker**	1.3 mm		
**Hypertension**	8 (47%)		
**Genetic occurrence**	6 (35.3%)		
	1 (5.9%)		
**Multiple aneurysms**	2 (11.8%)		

Mean ± standard deviation (minimum–maximum) or absolute number of cases (relative frequency in %).

**Table 2:** Results characteristics.

Results			
***n* = 17**	RROC 1	RROC 2	RROC 3
**Immediately after treatment**	13 (76.47%)	4 (23.52%)	0 (0%)
**6-month follow-up results**	14 (82.35%)	3 (17.65%)	0 (0%)

Absolute number of cases (relative frequency in %).

## Abbreviations

SAC	stent-assisted coiling
mRS	modified Rankin Scale
DSA	digital subtraction angiography
MCA	middle cerebral artery
ICA	internal carotid artery
BA	basilar artery
RROC	Raymond-Roy occlusion classification
